# Foot skin depots of ^18^F-fluorodeoxyglucose do not enable PET/CT lymphography of the lower extremity lymphatic system in man

**DOI:** 10.1186/2191-219X-3-17

**Published:** 2013-03-14

**Authors:** Mads Radmer Jensen, Lene Simonsen, Markus Lonsdale, Jens Bjørn Bülow

**Affiliations:** 1 Department of Clinical Physiology and Nuclear Medicine, Bispebjerg Hospital, Bispebjerg Bakke 23, Copenhagen, NV 2400, Denmark; 2 Department of Biomedical Sciences, The Panum Institute, University of Copenhagen, Blegdamsvej 3B, Copenhagen, N 2300, Denmark

**Keywords:** FDG, Sentinel lymph node, Lymphoscintigraphy, Intradermal depots, PET/CT imaging

## Abstract

**Background:**

In mice, ^18^F-fluorodeoxyglucose (^18^F-FDG) positron-emission tomography/computed tomography (PET/CT) lymphography enables detailed imaging of the lymphatic system and quantification of lymph node function. If this applies to humans, it may improve staging of several malignancies. The aim of this study was to elucidate whether foot skin depots of ^18^F-FDG make PET/CT imaging of the lower extremity lymphatic system possible in man.

**Findings:**

In four healthy volunteers, ^18^F-FDG depots (5 MBq in 0.1-mL isotonic saline) were injected intradermally in one foot and subcutaneously in the other. Activity was measured in blood samples drawn simultaneously from the great saphenous veins about 5 cm proximal to the ankle joints and a medial cubital vein before and every minute for 15 min after depot injection. Immediately thereafter, a low-dose CT was performed from the ankles to the pelvis followed by two consecutive PET scans of the same region.

Blood activity increased faster and to a greater extent in the great saphenous veins compared to the medial cubital vein. PET/CT images showed activity in the superficial and deep veins of the lower extremities. No lymphatic collectors or nodes were visualized.

**Conclusion:**

Neither subcutaneous nor intradermal injection of ^18^F-FDG allows imaging of the lower extremity lymphatic system in man.

## Findings

### Background

Sentinel lymph node biopsy plays a key role in the staging of malignant melanoma [1] and breast cancer [2]. Conventionally, sentinel lymph nodes are localized by γ-camera imaging after injection of ^99m^Tc-labeled colloids. During surgery, γ-probe counting and peritumoral blue dye injections guide sentinel lymph node resection [3]. However, these techniques have received critique for being time-consuming and having poor spatial resolution [4].

In a recent murine study, it was shown that intradermal injection of ^18^F-fluorodeoxyglucose (^18^F-FDG) into the tail skin enables detailed imaging of the lymphatic system and quantification of lymph node function by means of combined positron-emission tomography/computed tomography (PET/CT) [4]. In that study, it was suggested that these findings might apply to man. If so, it may improve the sentinel node technique through increased spatial resolution, shorter acquisition times, and improved anatomical localization.

However, in man, a likely path for the removal of intradermal depots of ^18^F-FDG is through diffusion to systemic capillaries. Activity in the blood from local veins will increase faster and to a larger extent compared to that in the blood from distant veins, and PET/CT imaging will show activity in the local veins. Contrarily, if ^18^F-FDG is drained primarily via the lymphatic system, a delay is expected before ^18^F-FDG activity increases in venous blood due to the transit time through the lymphatic system, and PET/CT imaging is expected to visualize lymphatic collectors and lymph nodes.

The aim of the present study was to elucidate whether foot skin depots of ^18^F-FDG allow for PET/CT lymphography of the lower extremities in man.

### Ethical approval

The Science Ethics Committees for the Capital Region of Denmark approved the study (protocol number: H-2-2012-162). Written informed consent was obtained from all subjects.

### Subjects

Four subjects, three men and one woman, aged 33 to 62 years, participated as healthy volunteers. None of the subjects suffered from any known disease or took any medications. Small lower leg superficial venous ectasias were allowed in the female subject.

### ^18^F-FDG-blood activity

Each subject was placed comfortably in supine position on the scanner table. The great saphenous veins were catheterized about 5 cm proximal to the ankle joints, and a medial cubital vein was catheterized as well (20 gauge, BD Venflon™ Pro, Becton Dickinson Infusion Therapy AB, Helsingborg, Sweden).

Depots, each consisting of approximately 5-MBq ^18^F-FDG in 0.1-mL isotonic saline, were injected into the skin of the first toe interstitium on the dorsum of the feet using a 1-mL syringe and a 27-gauge needle. On one foot, the depot was injected subcutaneously and on the other intradermally. Intradermal injection was verified by the elevation of a wheal at the injection site. Careful aspiration before subcutaneous injection avoided accidental intravenous injection. Different depot placements were used to test whether this affects the drainage route (veins vs. lymphatics).

Blood samples (2 mL/sample) were drawn simultaneously from each catheter before depot injection and once every minute for 15 min thereafter. Samples were transferred to pre-weighed plastic tubes, and sample activity was counted in a well counter with decay correction (Wallac 1480 Wizard® 3”, PerkinElmer, Waltham, MA, USA). Finally, the samples were weighed (precision 1/100 g), and measured sample activity was corrected for sample weight.

One subject deviated slightly from the described protocol on the following points: (1) catheterization of the great saphenous veins only succeeded unilaterally (intradermal depot); (2) depot activities were approximately 1 MBq/depot.

### ^18^F-FDG PET/CT imaging

Immediately following the last blood sample, approximately 20 min after depot injection, without moving the subject on the scanner table, the PET/CT acquisition was started (Gemini TF®, Philips Healthcare, Best, The Netherlands). A low-dose CT (20 mAs/slice) was performed from the ankles to the anterior superior iliac spine and followed by two identical PET scans (1 min/bed position, axial coverage 18 cm, 50% overlap) of the same region. Protocol duration was approximately 30 min. PET images were reconstructed with the standard vendor-supplied ‘WholeBody’ protocol including corrections for decay, scatter, and attenuation and utilizing time-of-flight information.

### Results

**Figure 1:**
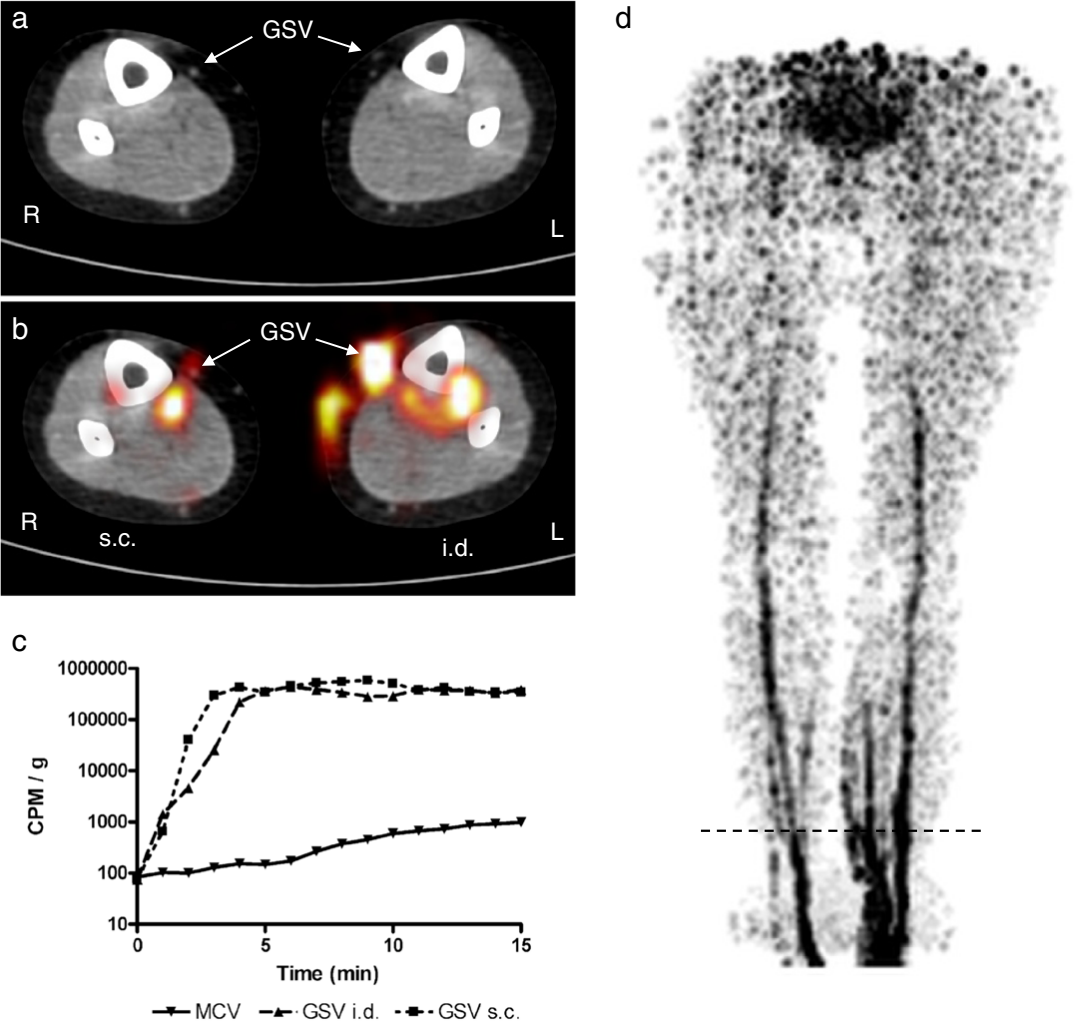
**Results for subject 1. **A 58-year-old woman. ^18^F-FDG (5 MBq in 0.1-mL isotonic saline) was injected into the dorsal skin of each foot subcutaneously (s.c.) on the right and intradermally (i.d.) on the left. (**a**) Axial low-dose CT of the distal lower leg. The great saphenous veins (GSV) are indicated by arrows. Scan level corresponds to the horizontal dashed line in (**d**). (**b**) Axial PET superimposed on CT showing ^18^F-FDG activity in a deep vein in the right lower leg. In the left lower leg, ^18^F-FDG activity is visible both superficially in the GSV and deeply in a posterior tibial vein with a communicant vein. (**c**) Time-activity curve for blood samples from the medical cubital vein (MCV) and GSVs with either i.d. or s.c. depot placement. Note the logarithmic scale on the *y*-axis due to large differences in measured activity. (**d**) Thick-slab maximum-intensity-projection PET image of the lower body showing the activity distribution of ^18^F-FDG.

**Figure 2:**
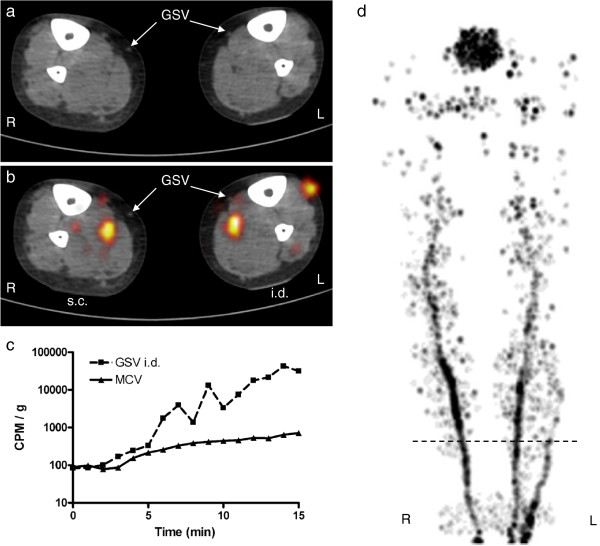
**Results for subject 2. **For figure details, see legend of Figure 1. A 62-year-old male. Injected depot activity was 1 MBq in 0.1 mL/depot. Catheterization was only possible on the left leg (i.d. depot). On the right lower leg (s.c. depot), ^18^F-FDG activity is visualized in a deep vein, while on the left lower leg, activity is visualized both in a deep and a superficial vein.

**Figure 3:**
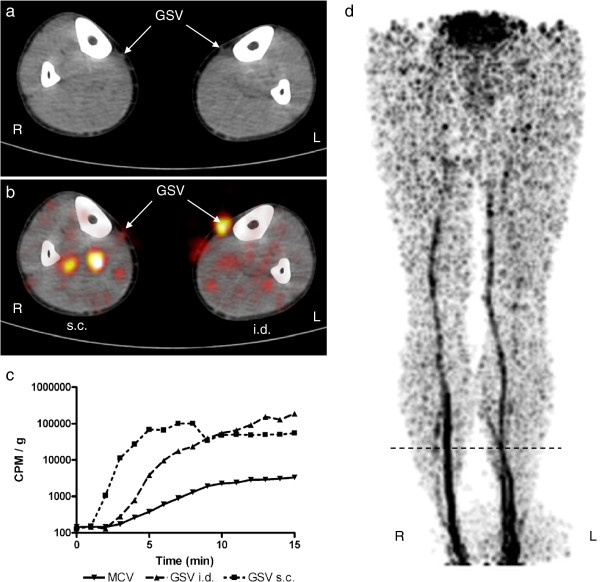
**Results for subject 3. **For figure details, see legend of Figure 1. A 45-year-old male. On the right lower extremity (s.c. depot), ^18^F-FDG activity is visualized in deep veins, while on the left (i.d. depot), in the great saphenous vein.

**Figure 4:**
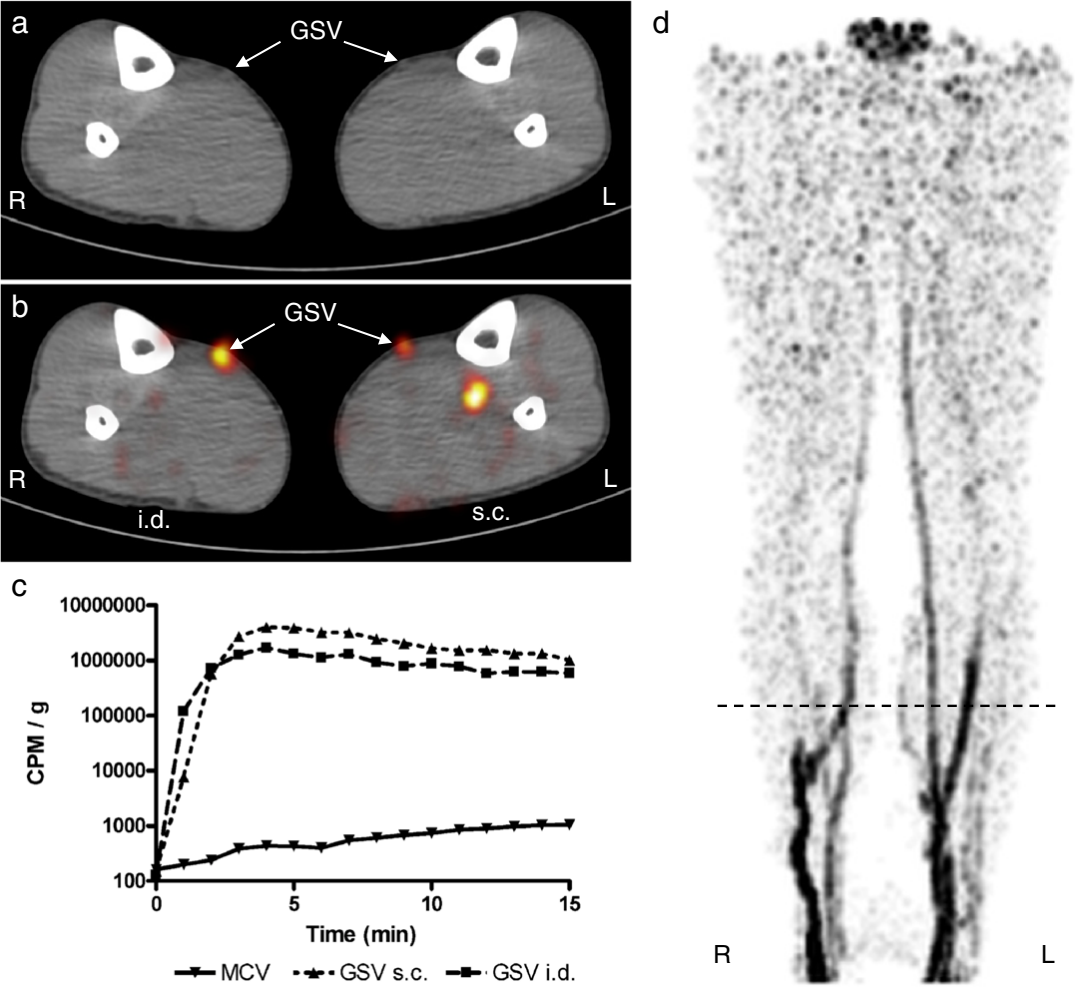
**Results for subject 4. **For figure details, see legend of Figure 1. A 33-year-old male. ^18^F-FDG activity is visualized in the great saphenous veins bilaterally, but on the left (s.c. depot), also in a deep vein.

The results for each subject are shown in Figures 1, 2, 3, and 4. Fused PET/CT images revealed ^18^F-FDG accumulation corresponding to both superficial and deep veins of the lower extremities in all subjects. Venous drainage routes showed both intra- and interindividual variation. Drainage pattern was not linked to depot placement. Neither lymphatic collectors nor lymph nodes were visualized in any subject.

Time-activity curves showed that activity in great saphenous vein blood increased faster and to a greater extent compared to medial cubital vein blood in all subjects. Great saphenous vein blood activity exhibited similar patterns for intradermal and subcutaneous depots.

### Discussion

In mice, ^18^F-FDG positron lymphography provides detailed visualization of lymphatic collectors and lymph nodes as well as quantification of lymph node function after intradermal injection into the tail skin. However, we show that this finding cannot be extrapolated to humans as both intradermal and subcutaneous depots of ^18^F-FDG cause significant and immediate activity accumulation in local venous blood. Both fused PET/CT imaging and time-activity curves showed this.

In man, transcapillary transport of glucose is governed by diffusion through the interendothelial junctional space [5]. A skin depot of ^18^F-FDG gives rise to a large local concentration gradient between the interstitium and capillary blood facilitating passive washout of the depot to the systemic capillaries. Our results underline that important physiological differences exist in ^18^F-FDG transport between mouse tail skin and human lower extremity skin. One mechanism, which can explain the species difference, is that the mouse tail skin is more tightly bound than the skin on the human foot. This results in a higher hydrostatic pressure in the ^18^F-FDG depot in the mouse tail. It is well described that a high interstitial pressure promotes lymphatic drainage [6]. Another possible mechanism for lymph node uptake of ^18^F-FDG in the study by Thorek et al. is an inflammatory reaction to the co-injection of isosulfan blue with ^18^F-FDG. Although not common, it is well described that isosulfan blue injections in sentinel lymph node identification can cause skin reactions [7].

In lymphoscintigraphy, ^99m^Tc is coupled to larger molecules such as human colloidal albumin to avoid capillary washout and thus facilitate drainage by the lymphatic system [8]. We suggest that a similar principle needs to be applied in PET/CT lymphography in order to make the method feasible in man.

### Conclusion

Lower extremity PET/CT lymphography using ^18^F-FDG skin depots in the feet is not possible in man due to significant tracer washout to systemic capillaries.

## Competing interests

The authors declare that they have no competing interests.
